# On Docking, Scoring and Assessing Protein-DNA Complexes in a Rigid-Body Framework

**DOI:** 10.1371/journal.pone.0032647

**Published:** 2012-02-29

**Authors:** Marc Parisien, Karl F. Freed, Tobin R. Sosnick

**Affiliations:** 1 Department of Biochemistry and Molecular Biology, University of Chicago, Chicago, Illinois, United States of America; 2 Department of Chemistry, University of Chicago, Chicago, Illinois, United States of America; 3 Computation Institute, University of Chicago, Chicago, Illinois, United States of America; 4 The James Frank Institute, University of Chicago, Chicago, Illinois, United States of America; 5 Institute for Biophysical Dynamics, University of Chicago, Chicago, Illinois, United States of America; University of South Florida College of Medicine, United States of America

## Abstract

We consider the identification of interacting protein-nucleic acid partners using the rigid body docking method FTdock, which is systematic and exhaustive in the exploration of docking conformations. The accuracy of rigid body docking methods is tested using known protein-DNA complexes for which the docked and undocked structures are both available. Additional tests with large decoy sets probe the efficacy of two published statistically derived scoring functions that contain a huge number of parameters. In contrast, we demonstrate that state-of-the-art machine learning techniques can enormously reduce the number of parameters required, thereby identifying the relevant docking features using a miniscule fraction of the number of parameters in the prior works. The present machine learning study considers a 300 dimensional vector (dependent on only 15 parameters), termed the Chemical Context Profile (CCP), where each dimension reflects a specific type of protein amino acid-nucleic acid base interaction. The CCP is designed to capture the chemical complementarities of the interface and is well suited for machine learning techniques. Our objective function is the Chemical Context Discrepancy (CCD), which is defined as the angle between the native system's CCP vector and the decoy's vector and which serves as a substitute for the more commonly used root mean squared deviation (RMSD). We demonstrate that the CCP provides a useful scoring function when certain dimensions are properly weighted. Finally, we explore how the amino acids on a protein's surface can help guide DNA binding, first through long-range interactions, followed by direct contacts, according to specific preferences for either the major or minor grooves of the DNA.

## Introduction

Interacting molecules convey information via their association that is driven by surface complementarity and chemical compatibility. However, predicting the docking process generally requires some structural knowledge (although attempts exist of simultaneously predicting folding and docking [1]). Given a number of recent and promising structure prediction algorithms for DNA [2], RNA [3], [4], [5], and proteins [6], [7], along with the wealth of data generated by various structural genomics initiatives [8], [9], a major goal is to devise methods for the automatic determination of gene and protein networks at a molecular level and on a genomic scale. This daunting task requires docking algorithms that can handle the three major classes of molecules, as well as large scale computing resources to perform the computations on a genomic scale.

Although a number of approaches are available for predicting the docking of small ligands and proteins with other proteins (although not necessarily highly successful), the treatment of the docking of nucleic acids (NAs) onto proteins lags far behind. The continually growing database of solved structures for NA-protein molecular complexes [10] is available for mining to extract the rules governing molecular association [11], [12], [13]. As found in the PDB, the protein-protein docking process is the most studied and critically reviewed [14], [15], [16] because it is believed to be the most prominent molecular assembly [17]. The fewer existing studies for protein-NA docking [18], [19] include those with DNA [18], [20], [21] and RNA [19], [22] and with force field development [23]. These studies mainly employ scoring functions that either involve all the atoms or all beads in reduced many bead models, but the scoring functions are meaningful only in assessing the structure of a solved complex or a very high resolution models. Other treatments use fuzzy restraints, for example, from experimental studies [18] but are restricted to treat either protein-DNA or protein-RNA interactions.

Our goal is two-fold. The first goal is to analyze the behavior of the rigid body docking methods when beginning with either the bound (apo) or unbound (holo) crystal structures for the molecules to be docked. The second goal is to assess the performance of two published statistical scoring functions, commonly known as statistical potentials, and to compare them with our new statistical potential that is based on machine learning methods and that contains orders of magnitude fewer parameters than the published statistical potentials. The study further aims to provide reasonable performance using only the holo forms of the molecules and to devise scoring functions that are able to discern credible or native-like docking poses.

## Results and Discussion

### Protein-DNA benchmark

We use van Dijk and Bonvin's protein-DNA benchmark set composed of 47 complexes whose PDB IDs are summarized in Table S1 [24]. This benchmark includes the bound and unbound forms of the proteins and DNAs, enabling tests of the importance of flexibility and conformational change for both the protein and DNA as well as on the performance of rigid body docking methods and various scoring functions.

### Performance of rigid body docking

Rigid body docking methods have garnered tremendous attention because of the development of fast geometric algorithms using fast Fourier transform calculations of surface complementarity [25]. These methods can be systematic and exhaustive in the exploration of all degrees of freedom since 10^10^ docked conformations or poses can be generated. But the analysis of such a large decoy set is challenging. Hence, most applications including ours use far fewer poses.

We retain the best 10^5^ models from the entire 10^10^ models produced for each protein-NA complex by FTdock [26], [27] and query whether such a large decoy set is required to find a sufficiently accurate pose. The best models are initially selected based on surface complementarity scores (SCS) assigned by FTdock, rather than on chemical and electrostatic compatibility. These models are assigned an SCS rank from 1 to 10^5^. Models also are ranked by the root-mean-squared-deviation (RMSD) calculated between the model and native DNA conformations after the proteins have first been aligned.

In order to further restrict the size of the decoy set, only poses with SCS ranks below a threshold (e.g., the top 10^3^ decoys from the 10^5^ set) are retained. The degree of success is defined as the fraction of the best 20 RMSD scoring decoys in this smaller subset. This procedure allows us to examine whether the subset of decoys with the best surface complementarity also contains poses with low RMSD to the native pose.

**Figure 1:**
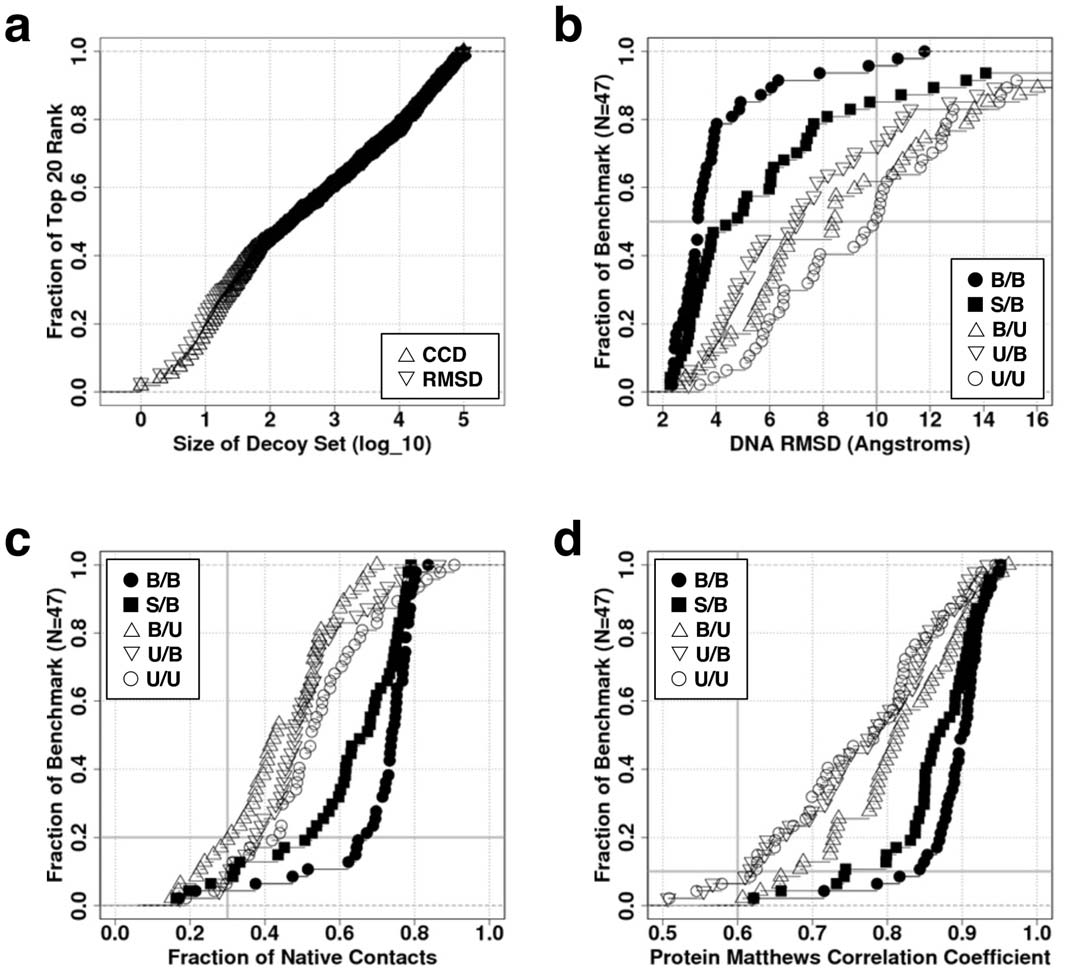
Performance of rigid-body docking. (a) Justification for decoy set size. The top 20 CCD (▵) or RMSD (▿) ranks for the N = 47 complexes in the benchmark set are plotted against the size of the decoy set generated using molecules in the bound conformation. (b,c,d) The performance for the five docking trials using different combinations of bound and unbound conformations: bound protein/bound DNA (•, B/B), bound, but with rebuilt side chains (▪, S/B), unbound/bound (▿, U/B), bound/unbound (▵, B/U), and unbound/unbound (○, U/U). A total of 10^5^ docked conformations are generated for each complex. (b) Fraction of the complexes having an RMSD of the 20^th^ best decoy better than the abscissa (e.g., 50 percent of the benchmark can be rebuilt to within 10 Å using the unbound forms as marked with the two intersecting grey lines). (c) Same as (b) but for fraction of native contacts. The higher the score the better; docking would have the value 1.0. For 80 percent of the benchmark, decoys have at least 30% of the native contacts (intersecting grey lines). (d) Same as (b) but for the MCC. The true positives (TP), false positives (FP) and false negatives (FN) are computed by comparing the protein residues in contact with DNA in the crystal structure compared to a docked model. Higher values are better; a perfect docking would score1.0. As much as 90 percent of the benchmark decoys have MCC greater than 0.6.

Figure 1a presents the fraction of the 20 lowest ranking RMSD in the decoy subset as a function of the size of the decoy subset. Here, we use the bound forms of the molecules because they provide for a mimic of a best case scenario. On average, 12 of the best 20 RMSD poses are found in the subset of the best 10^3^ decoys that have been selected according to SCS rank. This number increases to 16 when the subset contains the best 10^4^ SCS decoys, and, of course, increases to 20/20 in the entire set of 10^5^ decoys. Although the increase in low RMSD structures is mild, it does indicate that on average models can still have low RMSD even when the surface complementary is far from optimal. Nevertheless, we find that for some complexes, the entire set of 10^5^ poses is required to find low RMSD poses (data not shown). Consequently, we use 10^5^ poses in the following tests.

Next, we inquire how well rigid body docking describes protein-DNA interactions. The algorithm is challenged with five docking trials that utilize the four combinations of the bound and unbound conformations of the protein and DNA molecules, as well as an extra trial using the bound form for the DNA and protein but with the protein's side chains rebuilt using the SCWRL algorithm in order to study contributions from side chain reorganization. For each trial, 10^5^ decoys are generated for each of the N = 47 complexes, yielding a total of 23.5×10^6^ decoys. Three goodness-of-fit metrics quantify the performance: 1) The RMSD between the DNA molecules of the native and model once the proteins are aligned. 2) The fraction of native contacts, as defined in the CAPRI experiment [15]. A contact is defined to exist between a protein moiety and one in DNA if their separation is less than 7 Å. The intersection of the set of contacts in the native co-crystallized complex with the corresponding set in a model defines the true positives (TP), i.e., the predicted contacts in the model complex that are also present in the native complex. The difference between the sets represents the false negatives (FN), i.e., the contacts present in the native complex but absent in the model. The fraction of native contacts is then TP/(TP+FN). 3) The protein Matthews correlation coefficient (MCC) compares the set of protein residues in contact with DNA in the native complex with that of a model as follows. The native set comprises the residues in the complex found to be in contact with DNA, while those in contact with DNA in the model define the model set. The intersection between the sets gives the true positives (TP). Residues in contact in the native complex but not in the model are the false negatives (FN). Similarly, residues in contact in the model but not in the native are false positives (FP). The MCC is computed as (TP/(TP+FP) TP/(TP+FN))^1/2^. Because the MCC only considers the protein residues in contact with DNA, regardless of the DNA nucleotide, the MCC provides a less stringent than the measure of fraction of native contacts.

When the docked conformations of the molecules are used, a pose with a DNA RMSD≤4 Å is present for 80% of the N = 47 benchmark complexes (Fig. 1b). This result indicates that the rigid body docking procedure can be satisfactory. However, only about 50% of the benchmark complexes can be redocked to within 4 Å when the protein side chains are rebuilt using SCWRL but in the absence the DNA to reflect real world situations where only the protein's structure in the unbound state is known. Hence, the proper orientation of the amino acid side chains significantly influences the success of the docking. Our calculations use the default settings in FTdock to restrict the interpenetration of the two docked molecules; other settings may perform better. Here, it is tempting to compare the protein-DNA results to those of protein-protein docking predictions. Because of the elongated shape of the DNA molecule, any small tilt of the DNA significantly increases the RMSD, whereas proteins, with their more globular shape, show RMSD values that are more robust to misdocking. We suggest that the CAPRI criteria for pose quality should be revised to take this effect into consideration.

A sharp decrease in performance ensues when either or both of the molecules are in the unbound or holo state. Merely 5% of the systems achieve the threshold of RMSD^best^≤4 Å, while only 50% can be rebuilt to within 10 Å when using unbound conformations. Interestingly, the overall performance is slightly better when the protein is in the holo state. Potentially, docking using various DNA shapes could be a quick alternative to test a new target (Figure 1b, U/B: unbound protein/bound DNA). Regardless, these performances are underwhelming for the situation that best corresponds to a realistic scenario. One can find the data for Figure 1b in Table S6.

When the fraction of native contacts is taken as the measure of performance, 80% of the 47 benchmark complexes are rebuilt with 30% of the native contacts or better (Figure 1c). This value of 30% corresponds to the minimum at which the docking is designated as “medium” in the CAPRI competition. Strikingly, the performances are better using the unbound forms of both the protein and DNA; more than 90% of the complexes yield medium poses or better. This striking outcome may be quite significant in enabling the use of rigid body docking with the holo form when screening a structural database to find a suitable binding target, provided that the scoring functions are sensitive enough to identify these “early-encounter” conformations that form just prior to relaxation of the DNA and/or protein conformation. This phenomenon of improved docking with the unbound states is consistent with the “funnel-energy model” as reported in a recent study of protein-protein binding [28].

Similarly, up to 90% of the benchmark complexes yield protein MCC indices ≥0.6 when using the unbound structures (Figure 1d). Therefore, the use of the holo form in rigid body docking can still provide useful information as to where a DNA molecule may bind and thereby guide experimental studies. The next section describes a new measure to assess the quality of rigid body docking models.

### Physical representation of protein and NA

**Figure 2:**
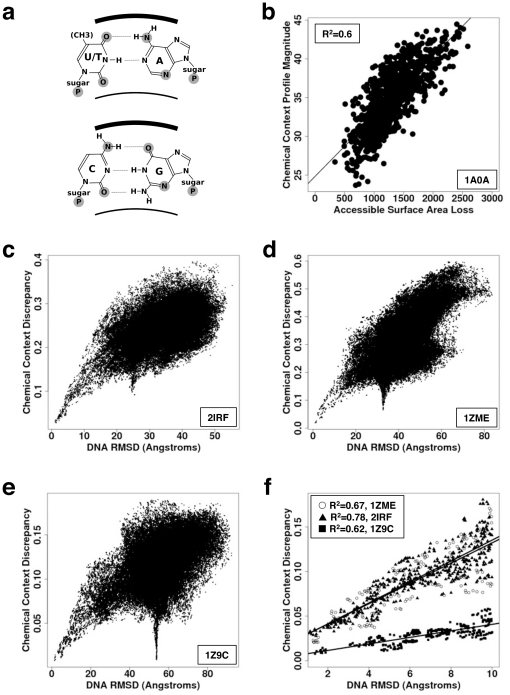
Protein and DNA representation and the Chemical Context Profile. (a) Interacting centers for nucleic acids. For each of the nucleic acids, A:adenine, C:cytosine, G:guanine, U:uracil and T:thymine, the three moieties used to describe a nucleotide are highlighted by shaded disks. Each nucleotide has three interacting centers; one in the phosphate group and one in each of the grooves, major and minor. The nucleic acids are paired in the canonical Watson-Crick configuration to expose the positions of the two double-helical grooves. The thick curved lines represent the major grooves (over the base pairs), while the thin ones the minor grooves (under the base pairs). (b) Comparison of the CCP magnitude with the loss of accessible surface area upon docking using complex 1A0A with a 10^3^ poses decoy set. The area loss is computed as the area of the complex minus the area of the isolated protein and DNA, using the msms computer program (ref). The squared Pearson correlation coefficient is 0.6. (c,d,e,f) CCD versus RMSD for 10^5^ decoys. The CCD correlates with the RMSD when the RMSD values are low (e.g. <10 Å). (c) The non palindromic DNA (2IRF). (d) The DNA sequence (1ZME) has two palindromic regions at both ends (e) The DNA (1Z9C) has a palindromic sequence. (f) Expanded version of (c,d,e) near the origin into the zero closed triangles, 2IRF; open circles, 1ZME; and closed boxes, 1Z9C. The Pearson's R-Squared values for linear fits are provided.

Our scoring function employs a representation describing the pairs of interacting chemical moieties between proteins and DNAs. The typical interactions are mostly polar, with the protein using positively charged side chains to “pierce” the electronegative envelope of the NA and to form a hydrogen bond with the base or sugar [29], [30], [31]. Since most of the interactions involve the phosphate backbone or sugar group atoms [13], [32], our scoring function uses one pseudo-atom to represent each of these interacting groups (Figure 2a). The backbone pseudo-atom is located at the position of the phosphorus atom (P). Because base pairs are recognized by different amino acid side chains in a manner depending on the base pair type and on access to the sugar [31], [33], [34], [35], each base is assigned two interacting centers (i.e., pseudo-atoms), one on the major groove side (M) and the other on the minor groove (m). This representation involves a total of 15 interacting centers to model the NAs (three moieties times five nucleotides).

Each of the 20 amino acids is represented by a single pseudo atom that is centered on the C_β_ atom because this position conveys most of the interacting information [36], and side chain information need not be specified, thus, better mimicking real world situations. Hence, the representation contains a total of 20 amino acids x 15 nucleic acids moieties, or 300 different pairwise interacting types. The next sections provide an examination of whether a rather small subset of the pairwise interactions can be extracted to suffice for our purposes.

### Chemical Context Profile

Supervised learning tasks must train the learning algorithm on objects that are defined as “good”. Identifying what is good for protein-NA docking becomes a non-trivial problem. Using the RMSD as a measure of goodness can mislead the learning algorithm, especially when many degenerate solutions of equal merit have different RMSDs. We define merit or success based on finding docking interfaces with the similar number and type of contacts as present in the native pose, rather than just those with a low RMSD.

This situation contrasts with that for protein-protein and protein-ligand docking where the RMSD [37] is the *de facto* scoring standard for assaying prediction quality. The RMSD, however, is poorly suited for scoring protein-NA poses because NAs often have rotational and translational symmetries due to their double-helical structure. For example, a 180° rotation of a duplex with a nearly palindromic sequence produces a large RMSD, yet the pose still has a near-native set of contacts. Hence, this model should also be assigned a good score. Conversely, two DNA docking poses that have the same RMSD to the native pose can be of considerably different quality. One pose might preserve most of the chemistry (e.g., due to a small tilt of the DNA), while the other produces an entirely different set of interactions (e.g., a small sideways translation of the DNA). The situation can be even worse when the molecule has high order symmetry such as the near palindromic DNA example. Furthermore, the RMSD often fails to capture the essence of the docking, which is best viewed as a search for a given set of molecular contacts between moieties of particular chemical character.

Accordingly, we introduce a 300-dimensional vector termed the Chemical Context Profile (CCP, see Materials and Methods) to describe a docking configuration. Each dimension of the vector is the interaction energy associated with one of the aforementioned 300 pairwise protein-NA interacting pairs (energies to be defined below). This vector is intended to capture the chemical context of the interacting surface. The CCP also correlates with the loss of surface area upon docking (using decoy set with one thousand decoys) (Figure 2b), and, hence, provides a quicker alternative for evaluating the area of the binding site than from analytical algorithms [38]. Below we demonstrate that the CCP is useful in scoring the decoys as well.

### Energy function

We now describe the energy function to be used in conjunction with the CCP. Protein-NA interactions include long-ranged electrostatic energies. However, their effective range is an open issue [39]. The side chain of each amino acid type has an average extent beyond the C_β_ atom. Within this distance, the interaction energy is assumed to be constant (Table S5), while beyond this distance, our potential decays as 1/*r*, similar to the electrostatic potential, with the specific functional form *f*(*r*) = 1/max(3.5,*r*-<e>), where *r* is the separation (in Å).

A possible concern about the long-range 1/*r* interaction is allayed by the fact that the Onsager length is 140 Å in a medium of low dielectric permittivity ε = 4, such as that inside a biomolecule. The binding interface between the protein and the nucleic acid is assumed as being largely desolvated. Because the rigid body docking approach only generates approximate models, we anticipate that the 1/r dependence should be adequate for our study.

### Chemical Context Discrepancy

The Chemical Context Discrepancy (CCD) is a measure introduced to guide the learning algorithm in selecting decoys with a native-like chemical context rather than a low RMSD. To emphasize our point, consider three DNA duplexes of increasing palindromic character. This increase results in the growth of a second minimum at ∼25 Å in plots of the CCD versus RSMD (Fig. 2c–e). A learning algorithm that is trained only on low RMSDs decoys would exclude those at 55 Å even though both configurations have essentially the same chemical context. Although a low CCD score does not imply a low RMSD, the converse is largely true, and the correlation is linear (Fig. 2f).

Because we seek to score based on poses having a native-like chemical context, docking configurations are considered as native-like when they have a similar CCP as the native pose. The difference in CCP profiles of the decoy and the native is quantified by the angle between the CCP vectors for the native and decoy poses, as determined using the dot-product 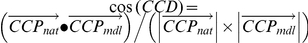. The smaller the angle, the more similar is their chemical context at the docking interface. The CCD has the advantage of being free of additional conditionals, whereas the RMSD and other measures of docking quality, such as the fraction of native contacts, depend on the labeling and mapping of moieties between the native and model poses. Another advantage of the CCD lies in the fact that discrepancies are implicitly weighted by the magnitudes of the entries in the CCPs. The CCD serves to identify the characteristics of a good pose in the machine learning stage of our study.

### Training on known complexes

A frequently used scoring function is a statistical potential that is constructed from a database of protein-NA complexes, ideally utilizing an appropriate reference state [36], [40]. Unfortunately, training on a low number of complexes may produce unreliable statistics. This phenomenon becomes serious for the development of a distance dependent potential because the counts are diluted across multiple distance bins. Furthermore, a distance dependent statistical potential may be too sensitive to permit efficaciously sorting out native-like poses in decoy sets with medium resolution, such as those produced by rigid body docking of the unbound forms of the molecules. Statistical potentials often utilize hundreds of parameters, which results in a small data∶parameter ratio. The large parameter space detracts from the learning capacity and may result in a learn-by-heart situation (i.e., over-fitting). We, therefore, seek to compress or limit the number of free parameters in our scoring function so as to produce a superior and quicker scoring function.

Our CCP vector offers a straightforward solution for training based on known complexes. By weighting each one of the 300 interacting pair types in the CCP, a linear combination of the entries in the CCP vector serves as the dominant term in our scoring function, 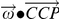. The weight 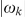 assigned to a given interacting pair of type *k* is the importance of this particular interaction in influencing the score. These interactions implicitly encompass factors such as electrostatics, hydrogen bonding, desolvation, London dispersion, polarization, ionization, and other effects. The 1/*r* dependence of our energy function mimics the function form of Coulomb's law although we do not explicitly account for the individual contributions of these factors to the total score, nor do we assume that the various weights can be uniquely decomposed into discreet components (as in Coulomb law's dependence on the product of charges Q_1_Q_2_). The sign of the weight indicates whether the interaction is attractive (negative) or repulsive (positive).

Table 1 displays the components of the compressed parameters space. These constitute the weight vector ω. The total score S is defined as 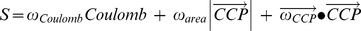, where Coulomb is the total 1/*r* Coulombic energy between the negatively charged phosphates with the positively and negatively charged amino acids. Without loss of generality, the contribution from the total Coulomb interaction has a weight of 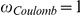.

**Table 1:** Scoring function components optimized in this work.

Attractive		Repulsive	
ASP-P	−1.2437400	ALA-P	+1.8794400
LYS-P	−0.6089050	PHE-P	+1.1204700
GLN-P	−0.6009340	TRP-M	+0.4848890
THR-P	−0.0755443	PHE-M	+0.0466844
CYS-M	−1.0494700	PRO-m	+0.6295210
THR- M	−0.6104230	CYS-m	+0.5091100
TYR-m	−0.6844790	GLN-m	+0.1395020
ILE-m	−1.0006000		
|CCP|	−0.3550520		

Attractive and Repulsive		
Coulomb	+1.0000000	ARG, LYS, HIS: +1e
		ASP, GLU, P: −1e

The components are split into three classes, depending on their sign: negative (attractive), positive (repulsive). The Coulomb potential is attractive or repulsive depending on the product of charges. Each component XXX-x is labeled with an amino acid, followed by a dash, then a nucleic acid moiety, which can be one of: phosphate group, P; major groove, M; and minor groove, m. The |CCP| component is the vector length of the Chemical Context Profile, which is proportional to the loss of accessible surface area upon binding. Pair-wise interactions selected by the Forward Sequential Feature Selection methods are in the form XXX-x.

Even though there are 300 different types of NA-protein pairwise interactions, we seek find a much smaller subset that can still identify native-like docking poses. As a first step, we no longer distinguish between the NA residue type for the score calculation (but the NAs remain distinct in the CCD calculation). Fifteen of the 17 components shown in Table 1 are selected via the Forward Sequential Feature Selection (FSFS) method from the 60 possible components (20 amino acids times the three moieties for DNA; Phosphate, Major and Minor grooves). Their weights, as well as 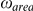, are determined via machine learning (below). The most attractive or repulsive entries are all associated with interactions involving the DNA's phosphate group. Hydrophobic side chains tend to stay away from the DNA. The weight 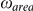 has a negative sign meaning that the larger the extent of the contact between the DNA and the protein the better the score. We prefer the use of |CCP| instead of FTdock's Surface Complementarity Score because the latter requires a Fourier transform. Hence, the full 300 dimensional CCP vector is used for assessing the quality of a pose with respect to the native solution via the CCD, while a reduced or compressed 15 dimensional CCP vector is use while scoring poses. The use of only 15 dimensions (i.e. number of fitted parameters) for scoring prevents over fitting. Indeed, Figure S2a shows that the scoring performance on the training set can still be improved by the addition of even more parameters but at the cost of diminishing test set performance (when the issue of over fitting starts to become significant).

### Machine learning

**Figure 3:**
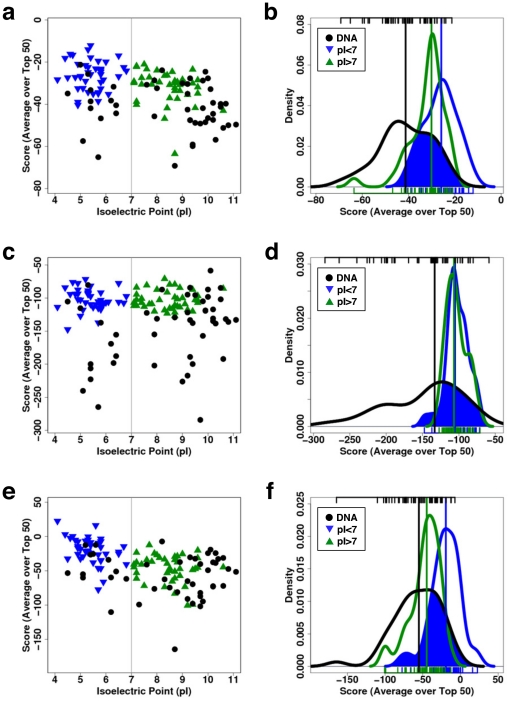
Performance of various scoring functions for identifying DNA binding. The scoring functions are statistical contacts (a,b), statistical distance-dependent (c,d) and the one derived here (e,f, bottom row). Three decoy sets are used, known DNA-binding proteins (•), proteins with isoelectric points (pI) lower than 7 (▿) and greater than 7 (▵). Left panels (a,c,e) illustrate how the protein-DNA complexes are scored in relation to the pI of the proteins. Right panels (b,d,f) illustrate how the scores of the three decoy sets overlap with one another: a perfect scoring function would be able to systematically score authentic DNA-binding proteins from those that do not bind to DNA (no overlap). The blue region highlights the overlap between the known DNA-binding proteins with those proteins that have a pI greater than 7. The tick marks at the top of the plots indicate the scores of the known DNA-binding proteins, while those at the bottom are for the two other protein sets.

Our goal is to produce a general method to identify potential protein-DNA binding partners and possible docking conformations. CCP vectors are evaluated for each of the 10^5^ poses generated for each possible protein-DNA pair. Certain dimensions of this vector are more heavily weighted than others to produce a net score S (defined earlier) that is favorable only for likely protein-NA poses, with minimal false positives and negatives; i.e., the best weights vector ω is the one that maximizes the separation between the scores of known DNA-binding proteins from randomly chosen proteins from the PDB not known to bind DNA (e.g., Figure 3, left column).

These weights are determined using a training set with 34 complexes. For each complex, the 10^5^ decoys are first sorted by their CCDs, from smallest (most native like) to greatest. The best 50 decoys for each complex are identified using the scoring function S defined previously, producing the training set of 1700 decoys (50×34 complexes). The weight vector ω is adjusted to select for the most native-like among the 1,700 poses where the most native-like is determined by our CCD parameter which examines whether the chemical context of the pose is similar to that of the native pose (rather than, e.g., the RMSD). Specifically, we optimize the objective function,
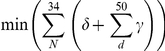
where 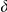 equals −100 if at least one decoy has a CCD rank lower than 100, while the function 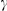 equals −1 if the decoy's CCD rank is less than 100. The ranking system allows for a uniformly weighted objective function, in contrast to the use of the CCD values themselves, which vary broadly depending on the size of the molecules (e.g., compare the y-axes in Figures 2c–e).

The optimization of ω includes proteins with both low (<7) and high (>7) isoelectric points (pI) because the net charge of the protein, either negative or positive, respectively, is a significant factor in identifying authentic complexes (Figure 3f). A potential improvement to our approach would be to train separately on the complexes with low and high pI separately, as each protein class may use a different balance of forces to bind DNA. When the pI is above 7, nonspecific electrostatic interactions could dominate and produce a high number of false positives. Hence, specific interaction should be given increased weight for this class of proteins, as compared to proteins having a pI below 7.

### Performance of scoring functions

Our method is now compared to two statistical potentials aimed at identifying protein-DNA complexes. The first approach only considers contacts [41], and the other only distances [42]. These potentials are challenged to answer the following questions. “Does this protein bind DNA, and if so, where on the protein would the DNA bind?” These questions may be difficult for the statistical potentials to answer considering the limited context in which they are developed. On the one hand, the contact statistical potential is used for further filtering after proteins have already been selected to contain DNA-binding motifs similar to ones found in solved DNA-protein complexes. On the other hand, the distance statistical potential is sensitive enough to predict binding affinities, mutation induced binding affinities, and native base pair recovery. These tests require protein-DNA templates with atomic precision but are advertised to perform docking decoy discrimination. We test these methods along with ours in a more stringent application where the predictions start from the 3D structures of the isolated protein and DNA.

The first challenge “Does this protein bind DNA?” is pursued using three sets of proteins. One set contains the 47 proteins in the benchmark set (Table S1), while the other sets contain proteins with a pI<7 (N = 40, Table S3) or pI>7 (N = 41, Table S4), respectively. Separating the proteins by their pI is important because proteins with higher pIs contain more electropositive residues on their surfaces and are, thus, more likely to bind DNA. Proteins from the benchmark are docked against their cognate DNA, while proteins from the two other sets are docked against a straight B-DNA double helix with the same sequence as the DNA of PDB file 1A74. The use of cognate DNA for the benchmark proteins enables establishing a ceiling for the ability of the various scoring functions to identify known DNA-binding proteins. If the scoring fails to identify cognate DNA, then there is little hope when a “generic” DNA is used.

Figure 3 (left column) illustrates how the three scoring functions behave with respect to variation of the protein's pI (Figure S1 for FTdock's Coulomb and Surface Complementarity Score). As expected, the scores for proteins with high pIs are better than for those with lower isoelectric points, except for the distance scoring function where the scores are similar (compare Fig. 3d with Figs. 3b, 3f). This similarity is probably due to the extremely large parameter space (167 atom-types for proteins times 82 atom-types for DNA, yielding individual parameters for the 13,694 possible pairs of the distance-dependent potential). Table 2 indicates that median Z-scores for both low and high pI proteins are approximately, +0.87 versus +0.84, when scoring using the distance-dependent potential. The influence of the pI on the Coulomb-only score is evident, as the high pI proteins score better than known DNA-binding proteins (negative Z-Score for “Coulomb” in Table 2). The contact version produces better Z-Scores, but the distance-dependent version yields minimal overlap between curves for the scores of known versus unknown DNA-binding proteins (Figure 3, right column). Furthermore, there is minimal overlap of the low and high pI sets with the known below average scoring DNA-binding proteins, and hence a protein with a score better (lower) than −150 is very likely a DNA-binding protein (i.e., generating few false positives). But, one must bear in mind that the trial DNA structures used as probes to measure the DNA-binding affinity are the cognate DNAs.

**Table 2:** Performance of various scoring functions.

Scoring Function	Z-Score	Z-Score	Area	Area
	pI<7	pI>7	pI<7	pI>7
FTdock SCS	+0.64	+0.70	0.48	0.40
Coulomb	+1.15	−0.02	0.52	0.83
C-S	+1.34	+0.84	0.47	0.58
D-S	+0.87	+0.84	0.42	0.41
This work	+1.22	+0.35	0.46	0.72

Scoring functions are FTdock's Surface Complementarity Score (SCS), electrostatics via Coulomb's law, a statistical potential based on contacts (C-S), a distance-dependent statistical potential (D-S) and our work. Each scoring function is used in scoring three sets of proteins; the set of N = 47 known DNA-binding proteins (set K), the set of proteins with pI<7, and the set with pI>7. From the distribution of scores on a given set, say set K, we obtain the average score μ and the standard deviation σ of scores: this in turn allows locating any scoring value χ following a Z-score computation Z = (χ−μ)/σ. We report here the Z-scores of the median scores on set pI<7 and set pI>7. Positive Z-Score values indicate that the median has a value greater than the mean of set K, while negative ones lower. Ideally, the mean of set K should be lower than the median of the other sets (i.e. set K has better scores). The greater the Z-Score the better is the scoring function at discriminating DNA-binding proteins from non-binders. Normalized overlapping areas are also reported.

**Figure 4:**
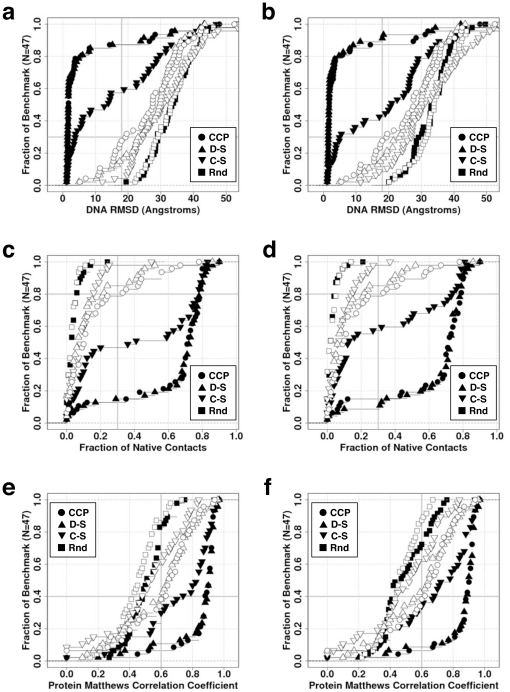
Performance of various scoring functions for identifying the native binding pose. The scoring functions are random (▪), statistical contacts (▾), statistical distance-dependent (▴) and our work (•). Two decoys sets are used for the evaluation: both protein and DNA in the bound form (closed figures) or both in the unbound form (opened figures). Left panes (ace) are results with the native DNA sequences, while the right panes (b,d,f) features “flipped” sequences. For each decoy set in the benchmark, the decoys are scored by the specified scoring functions (rows), then sorted from best to worst: lowest RMSD in top row, highest fraction of native contacts in middle row, and highest protein MCC in the bottom row, reporting the 5^th^ value in the top 50 score. (a,b) DNA RMSD. (c,d) Fraction of native contacts. (e,f) Protein MCC. The true positives (TP), false positives (FP) and false negatives (FN) are computed by comparing the protein residues in contact with DNA in the crystal structure compared to a redocked model.

The second challenge “Where would the DNA bind on the protein?” is addressed using the DNA's RMSD by considering the fraction of the native contacts reproduced and the Matthews correlation coefficient for the protein residues (Figure 4, rows). The challenge is performed with the cognate DNA sequences (left column) or the flipped DNA sequences (right column). A flipped DNA sequence has guanosine nucleotides substituted for adenosines (and vice versa) and cytosines for uracils (and vice versa). This flipping “mutation” retains the purine-pyrimidine order in the base pairs (i.e., a G = C base pair is flipped to an A = U pair). Each of the six plots in Figure 4 includes two sets of four curves: one set is for the bound/bound (filled symbols), and another set is for unbound/unbound states (opened symbols) of the protein/DNA molecules. Each set is examined with four different scoring functions; the three described earlier plus one called “random”, which simply attributes a random score to any docked complex. The random curves are important in providing a performance floor for comparison [43]. Because the analysis considers the top 50 scores in decoy sets of 10^5^ models, the statistical significance of a true positive hit (i.e., near native decoy identification) is high.

Both our scoring function and the distance-dependent function recognize native-like docking conformations when both the protein and DNA are in the bound forms, with 80% of the benchmark complexes having RMSDs below 5 Å (Figure 4a). Even though their curves are very similar, each scoring function makes distinct errors when identifying docking poses by score (Tables S7 and S8). Only about 40% of the benchmark is properly identified by the contact-only statistical potential at the 5 Å level. This result suggests that long-range interactions contribute to the recognition process. When the unbound forms of the molecules are docked, no scoring function is significantly better than random, at least when the DNA's RMSD is used to measure performance since 70% of the benchmark complexes have RMSDs above 18 Å. Surprisingly, when the DNA sequences are flipped, the contact-only potential performances are worst, which signifies that this statistical potential is more sensitive to the sequence context (Figure 4b). We expected to observe a diminished performance for the distance-dependent version, but this statistical potential is still able to choose native-like configurations even though the DNA sequence is not optimal.

Even if performance is assessed using less stringent criteria, such as the fraction of native contacts (Figures 4, middle row), all three scoring functions do not fare much better than random if both molecular states are holo; only 20% of the benchmark complexes have scores better than 0.3. The less stringent criterion, identifying the protein residues that contact the DNA, offers a more optimistic view of the performance of the various scoring functions. If we compare the unbound/unbound curve in Figure 1d, for which 90% of the complexes in the benchmark feature docked complexes with more than a 0.6 correlation, with the same curve in Figure 4e, as much as 60% of the benchmark still has a better than 0.6 correlation. On the one hand, this points to the usefulness of the rigid-body docking approach in identifying where the DNA would bind. On the other hand, these issues suggest that the quality criteria employed in the CAPRI test are probably too stringent for protein-DNA complexes to be attained with even with the best algorithms.

### Properties of DNA-binding proteins

**Figure 5:**
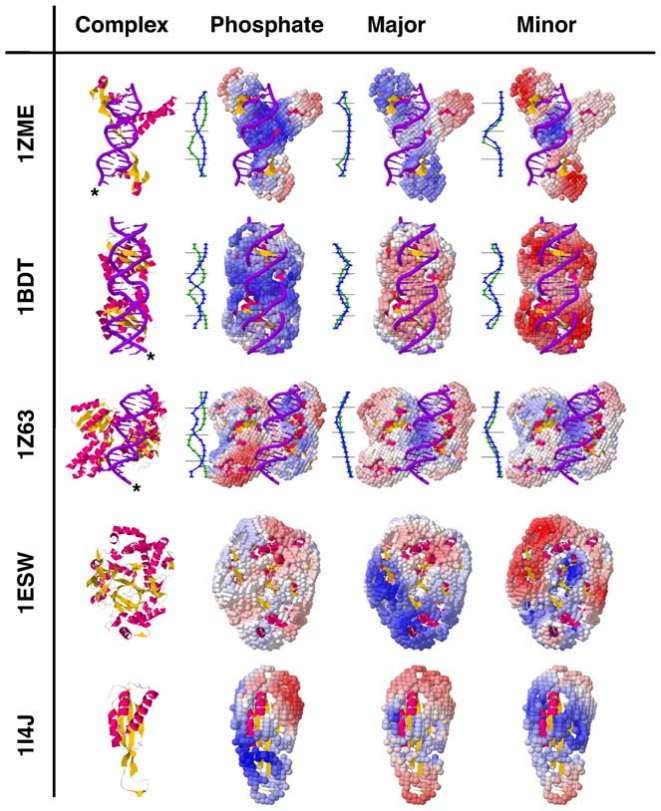
Protein DNA-binding potential. A protein's DNA-binding potential is revealed using one of the test moieties. Each test site is represented by a ball, whose color varies blue (favorable) to red (unfavorable). The first three proteins are known to bind DNA, while the last two are not known to bind DNA. For DNA-binding proteins, a graph tracks the potential for each type of moiety along the actual DNA coordinates. The blue curve tracks the 5′ strand of the DNA (tagged with a star), while the green curve tracks the 3′ strand. A point marks each base pair step, and a black horizontal line each 5 base pair steps. Since the 5′ phosphate is absent for both the 5′ and 3′ strands, the base pair step index starts at two. The potential is more favorable as the curve is more to the left. The x axis is scaled to show the relative change along the DNA molecule.

Because our interaction matrix only contains three types of moieties (phosphate, major and minor groves) within the DNA, probing a protein's surface with a “test” moiety enables highlighting the favorable and unfavorable regions for that test moiety, a procedure similar to the use of a test charge to probe an electrostatic field. Figure 5 displays the results of such tests for true DNA-binding proteins, as well as for proteins known not to bind DNA. The surface of each protein is scanned with either one of the test moieties, Phosphate, Major Groove or Minor Groove. The “Phosphate field” includes the contribution from the electrostatics of the protein. The moieties correspond to those illustrated in Figure 2a where each test site is represented with a ball whose color varies from blue (favorable) to red (unfavorable).

Each of the known DNA-binding proteins yields a favorable score for the Phosphate at the sites where the DNA docks the proteins (first three rows of Figure 5, second column). Furthermore, the Major and Minor Groove fields are complementary in 1ZME and 1BDT or overlapping in 1Z63. The two groove “fields”, along with the Phosphate field, align the protein onto the DNA with proper register. We hypothesize that the Phosphate field contributes an initial, indirect readout of the DNA by the protein, perhaps mediated by water [44], while the Major and Minor Groove measures function more as direct readouts once the protein-DNA interface is desolvated and scanned.

The three fields convey the “potential” for DNA binding, acting as a DNA-binding code [45]. Proteins known not to bind DNA present non-overlaping and discordant fields (last two rows of Figure 5), rendering them unable to coordinate and promote DNA binding. This test can be applied as a filter before a more computationally expensive docking procedure. It may also be used to explore how a protein can adapt for a competent DNA-binding field by a conformational change. These points are currently under investigation.

### Conclusion

There are five salient points in this study:

1. The introduction of the Chemical Context Profile (CCP) of the docking interface represents the chemical complementarity of the protein-NA interface for a given pose. The magnitude of the CCP correlates with the loss of surface area upon binding. Profiles from different poses can be compared with one another by their similarity to the native CCP, where the similarity is defined using the Chemical Context Discrepancy (CCD). Models with low RMSD to the native also have low CCD values, but the CCD measure is robust to DNA palindromic sequences. The CCD can be used as a substitute for RMSD in machine-learning techniques [46]. Furthermore, by weighting each entry of the CCP, we devise a simple and fast scoring function. The CCP is applicable to other types of interactions, including protein-protein, protein-RNA, protein-ligand interactions, etc. and the functional form is arbitrary (e.g., the alternative 1/r^6^). The CCP also can provide an estimate of the magnitude of various types of interactions (salt bridges, hydrogen bonding, hydrophobic desolvation, etc) provided that the entries in the CCP are designed accordingly.
2. The use of machine-learning techniques, more specifically sequential feature selection, can greatly reduce the parameter space in the derivation of a scoring function. This compression identifies the principal components of protein-DNA interactions. The compression process is implemented with a training set containing a small number protein-DNA complexes (N = 34), indicating that the method can be used when there are only a few structures. Very large decoy sets encompass many native-like complexes as true positive examples and provide far more true negative ones to score against. A total of only 15 components are identified as the principal components out of 300. This relative low number of parameters performs as well as a sophisticated statistical potential with several thousands of components, allowing for a quick complex scoring while preventing parameter over fitting.
3. Rigid-body docking is examined to see whether it can provide rudimentary “initial contact” protein-DNA docked complexes from the holo or unbound forms of both molecules. With a scoring function that is able to identify these complexes, rigid body docking can be used in all-atom refinement procedures that allow flexibility of both protein and DNA. These refinement procedures already exist, e.g., the HADDOCK approach [18], but this method requires near-native complexes for satisfactory results.
4. When the unbound states of both protein and DNA molecules are used, the performance of all the scoring functions tested herein is not much better than random. This situation likely reflects a “best case” actual genome-wide screening scenario because one or both structures generally are unknown. Hence, further research is required to increase the power of the scoring functions to identify early-encounter complexes. Unfortunately, no such structural database exists from which to extract any information. Potentially coarse-grain, Langevin or Brownian dynamics could be of use [47]. The least stringent criterion to evaluate the quality of docked poses is the one that measures the protein residues at the interface with DNA. Our scoring function performs comparably to modern statistical potentials and performs adequately in the identification of the surface residues involved in DNA binding. However, this performance is achieved using orders of magnitude fewer parameters.
5. We map a protein's surface to assess its potential to bind DNA. This ability is particularly important because electrostatic models of proteins are often inadequate to identify the DNA-binding properties of proteins [48].

## Materials and Methods

### Benchmark

An existing protein-DNA benchmark [24] consisting of 47 protein-DNA complexes is ranked from easy to difficult, depending on the degree of conformational change upon binding (Table S1). The benchmark provides the bound and unbound conformations, which enables testing the use of unbound forms of the molecules to rebuild a complex via rigid-body docking. The unbound forms of the proteins have been determined in the absence of their DNA partners, while the unbound forms of the DNA are generated by the computer program 3DNA [49] and adopt the standard B-form double-helix.

### RMSD

The root-mean-squared-deviation (RMSD) calculation includes the following heavy atoms: C_α_ and C_β_ for proteins and P, C2′ and C4 for DNA. All three DNA atoms appear in any nucleotide, regardless of type (A, C, G or T). The P atom is situated in the DNA backbone, C2′ in the DNA sugar ring, and C4 is in the nucleobase. This allows for easy computations of the RMSD between DNA molecules containing the same number of nucleotides but different sequences.

### Pose generation

The computer program FTdock [26], [27] is chosen to generate the docking poses. Even though it performs rigid body docking, FTdock has multiple advantages, including a systematic and exhaustive search of rotational and translational degrees of freedom with a bias toward geometrical surface complementarity [25]. FTdock also features a scoring scheme based on electrostatics [26]. We have modified the program to account for the unit negative charge on nucleic acid phosphate groups and have trivially parallelized the program to perform computations on a computer cluster without the need for communications between executing processes. We employ a 0.5 Å grid and 10° rotational spacing, yielding 15,840 possible rotation states. The best ten geometrical docking poses for each rotation are retained to give a grand total of 158,400 poses that are sorted by their surface complementary score (SCS), with the best 10^5^ retained. Because FTdock converts the molecules into coarse grids and allows for some overlap between them, it implicitly accounts for some induced fit upon docking. In the FTdock algorithm, one molecule is held in place while the other is scanned across its surface. The static molecule has a core in addition to an interfacial surface region. We choose the proteins to be the static molecule to account for side chain flexibility. The great number of decoys offers a reference state, similar in spirit to “decoys as a reference state” or DARS [50]. Hence, the scoring function has the ability to train on native-like docking poses and a great number of false positive docking configurations. Pose generation and management is made easy through the use of the Swift processing language [51], which can harness the power of super computers (University of Chicago's Beagle) as well as heterogeneous computer clusters (as those on the Open Science Grid).

### Chemical Context Profile

We define the Chemical Context Profile (CCP) as a multi-dimensional vector:
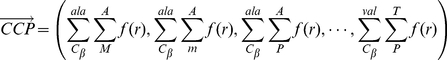
for which each entry is a double-sum over a given pairwise interaction type. The interaction is measured between specific moieties (bottom of the summation symbol) of specific residues/nucleotides (top of the summation symbol). For instance, the first entry is between all C_β_ moieties of the alanines (ala) in the protein, and major groove moieties (M) of the adenosines (A) in the DNA. The last term is between the valine (val) C_β_ and the thymine (T) phosphate groups (P). The moieties, or interacting centers, for proteins are centered at the C_β_ atom, while those for nucleic acids are presented in Figure 2a. For a given nucleotide, we only account for the residue interacting with the groove ((M)ajor or (m)inor) that is the closest to the residue's C_β_ atom, so that a residue interacts only with the closest groove. The total number of entries in the vector is 300: the 20 amino acid types times 3 NA moieties times 5 nucleotide types. The score is obtained by summing *f*(r) for each interacting pair, where the distance-dependent function is taken as *f*(r) = 1/max(3.5,r-<e>) and where the average extent of the amino acid side chains <e> are presented in Table S5. The functional form is designed to contain a maximum within each protein side chain's extent beyond which the interaction strength decays as the inverse of the distance, analogous to the decay of the electrostatic potential. The sum of the *f*(r) increases with the number of a given interacting pair type and with shorter separations.

### Pose scoring

Docked conformations are scored by weighting the Chemical Context Profiles, 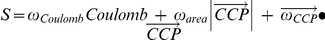, in which the dot symbolizes the vector dot product. The weights vector ω_ccp_ and ω_area_ are optimized using machine learning (as described below) to recognize native-like docking conformations in large sets of decoys. The weight 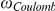 is set to unity without loss of generality. The |CCP| is a proxy for the area loss upon binding.

### Chemical Context Discrepancy

Given a native and predicted (model) docking pose and their associated CCPs, the Chemical Context Discrepancy (CCD) is defined as the angle between the profiles,
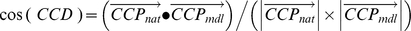
where the *nat* and *mdl* refer to native and model docking poses, respectively. The dot symbolizes the vector dot product, while vertical brackets indicate the vector norm, or magnitude. The CCD is used here as a substitute of the root mean squared deviation (RMSD) and is shown to correlate with low-RMSDs (Figure 2f) while taking into account the translational and rotational degeneracy of DNA molecules (especially those with palindromic sequences). Because moieties are triangulated by many others, it is almost impossible to generate two different docking poses for which their CCPs only differ by a constant. Hence, the CCD angle is zero if and only if the two CCPs are identical.

### Sequential Feature Selection

In order to identify the most important elements in an interaction matrix, tests of all subsets of the set of elements could be generated by enabling and disabling some of the matrix cells and determining how well the matrix performs at identifying native docking poses. This strategy would require 2^n×m^ parameter optimization trials for a matrix with *n* by *m* elements, repeated over many training and test sets (recall that the set of all subsets of a set of size N is 2^N^). Because our interaction matrix contains 20 * 3 * 5 entries, a total of 2^300^ experiments would be required. This number is computationally unfeasible even on a super computer. In practice, however, when the question is posed concerning a specific matrix (with many parameters), one can rely on the sequential feature selection (SFS) method [52]. Here, the parameters are activated in a sequential fashion, starting with one parameter (thus the forward version of sequential feature selection, i.e., FSFS). This process proceeds by activating all of the matrix features, one at a time. The feature that leads to the best performance when activated is permanently activated, and the selection process is repeated. We have performed the FSFS procedure for a matrix with 60 parameters that are associated with the twenty amino acid types and the grooves (M, m and P) of the DNA independent of nucleotide types (20×3 = 60 parameters). The FSFS is implemented to consider up to 30 parameters (Figure S2a). Although we explore only 30×60 subsets of the total 2^60^, this protocol is a tractable way to locate significant features or parameters. To derive the most important features, we simply count the number of times a feature appears after the feature selection round; those that are selected early appear more often as they are permanently activated, so they are deemed the most important. This step is performed to identify which elements in the scoring matrix have the most impact and, hence, require the most optimization (Figure S2b). A total of 15 variables have been selected by FSFS. Adding any more variables enhances the performance on the training set, but the performance on the test set starts decreasing (i.e., as the capacity increases, it is easier to learn the native docking poses by heart, but generalizing to new unseen cases becomes more difficult).

### Weight optimization

The weights are optimized using the Particle Swarm Optimization (PSO) method [53]. A variant of the PSO method is used to prevent early convergence and loss of diversity [54]. The PSO method entails evolving N = 50 particles in the space of parameters (our weights). The trajectory of each particle is controlled by its own local behavior as well as the current global minimum. The algorithm offers the advantage that the objective function being minimized has a unique global minimum, with few local minima, along a slice in the parameter space. Hence, whenever a particle updates the coordinates of its local minimum, we continue the optimization process in a stochastic fashion by choosing a random dimension, taking a step along this direction in both directions, and choosing the new position that further optimizes the objective function. The procedure is repeated until we fail to update the objective function ten times. To prevent the “learn by heart” over-training/fitting phenomenon, we rely on the early stoppage technique. After a fixed number of steps that is randomly chosen between 1–100, the optimization process is halted, and the current global minimum is reported as the solution.

The optimization program begins by loading the Chemical Context Profiles into memory for each of the decoys. The training set has 34 members (Table S2) and represents the N = 47 benchmark except for PDB codes 1Z– or higher, leaving these complexes for testing. 10^5^ decoys are generated for each member. The decoys are taken from the bound/bound states of the protein/DNA because they offer the best quality decoys with the lowest CCDs (or RMSD).

Given a weight vector ω obtained using the PSO, the score S of all decoys *d* is updated using the formula defined earlier. Then, the decoys are sorted by their scores from best (lowest) to worst (greatest) for each PDB ID. The best 50 decoys are identified and represent the docking set solution. Two objective functions discussed in the text are optimized. The best weights vector ω is the one that maximizes the separation between the known DNA-binding proteins and those proteins with low (<7) or high (>7) isoelectric points (Figure 3f). Entries of the best weights vector are shown in Table 1. A total of 200,000 weights vectors have been inspected for fits. The proteins with low and high isoelectric points are listed in Tables S3 and S4, respectively.

By grouping rows and columns together in the 20×15 interaction matrix, we can test many models for their ability to recognize native poses. For instance, one model has been identified by the Forward Sequential Feature Selection methods. Here, we considered the 20 amino acid types, and Major, Minor and Phosphate groups regardless of the nucleotide type for the nucleic acids (matrix size is 20×3 = 60). The selection process has identified 15 interacting pairs. Other models can readily be devised: the twenty amino acids could be grouped into six (as proposed here [55]). For the nucleic acids, we could consider the nucleotide types (A, …, T) (size = 5) regardless of the moiety type (M, m or P). Or, interactions with P can be specified as independent of the nucleotide type, while the Major and Minor are treated in a nucleotide-dependent fashion (size = 6; A,…,T,P). The model that performs best and has the lowest number of parameters is the one identified by the FSFS method. All other models either have more parameters with equal or worse performance.

## Supporting Information

Figure S1 **Performance of various scoring functions.** The scoring functions are FTdock's Coulomb's law (a,b) and Surface Complementarity Score (c,d). Three decoy sets are used: known DNA-binding proteins (•, black), proteins with pI<7 (▿, blue) and >7 (▵, green). Left panes show how the protein-DNA complexes are scored in relation to the isoelectric point of the protein. Right panes show how the scores of the three decoy sets overlap (blue area) with one another; a perfect scoring function would separate the scores of authentic DNA-binding proteins from those that do not bind DNA.
(PDF)

Figure S2 **Forward sequential feature selection.** The method is applied to find the most important interacting pairs. (a) As the number of variables increases, the performance on the training set (open circles) also increases, but the performance on the test set (closed circles) degrades when too many variables are used; this is the learn-by-heart phenomenon. We thus cap the number of variables to 17 (vertical grey bar); since two of the variables are fixed (Coulomb and |CCP|), 15 will be picked from the 20×3 = 60 interaction matrix. (b) The relative importance of the 60 pairs (left). The 15 most important pairs are highlighted in grey (right).
(PDF)

Table S1 **List of PDB codes that are part of the N = 47 protein-DNA benchmark database.**
(PDF)

Table S2 **List of PDB codes that are part of the N = 34 protein-DNA training set database.**
(PDF)

Table S3 **List of PDB codes that features N = 40 proteins with isoelectric point lower than 7, and are assumed to not bind DNA.**
(PDF)

Table S4 **List of PDB codes that features N = 41 proteins with isoelectric point greater than 7, and are assumed to not bind DNA.**
(PDF)

Table S5
**Amino acid extent beyond the** 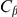 **atom.** Values extracted from 201 protein-DNA complexes. The magnitude reported is the average extent plus one standard deviation 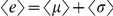. The PDB atom types used for the extent measurements are also shown.(PDF)

Table S6 **Performance of rigid body docking.** The performance is measured on a benchmark comprising 47 different complexes. Five docking trials using various combinations of bound and unbound states of the docked molecules: bound/bound (B/B), bound, but with rebuild side chains/bound (S/B), unbound/bound (U/B), bound/unbound (B/U), and unbound/unbound (U/U). A total of 10^5^ docked conformations are generated for each complex and docking trial. For each decoy set, decoys are sorted by RMSD from lowest to greatest, and the RMSD of the 20^th^ best decoy is reported. The RMSD is taken between the DNA positions in the native and the docked complexes, once the proteins have been superimposed. All values reported here are in Angstroms, and are plotted in Figure 1b. The Level column partitions the complexes into the Easy/Intermediate/Difficult notation assigned by van Dijk and Bonvin, which estimates the degree of conformational change upon docking.
(PDF)

Table S7 **Performance of various scoring functions.** The performance is tested using the bound states of the protein/DNA molecules. The scoring functions are our work (CCP), contact only statistical potential (C-S) and distance-dependent statistical potential (D-S). The RMSD column is the same as in table S6, B/B case. All values are in Angstroms, and are plotted in Figure 4a.
(PDF)

Table S8 **Summary of Tables S6 and S7, grouped by van Dijk and Bonvin's annotations.** A checkmark is put whenever the DNA RMSD is lower than 10 Angstroms. The FFT column relates to the ability of the FFT-based docking method to provide for good decoys, while the last three columns to the ability of the various scoring functions to identify them. Rows marked with an asterix have been left out while training, and as such constitute the test set.
(PDF)
