# Supplementary figures and images for: On Docking, Scoring and Assessing Protein-DNA Complexes in a Rigid-Body Framework
Source: PLoS One. 2012 Feb 29;7(2):e32647. doi: 10.1371/journal.pone.0032647 (PMC3290582; doi:10.1371/journal.pone.0032647)

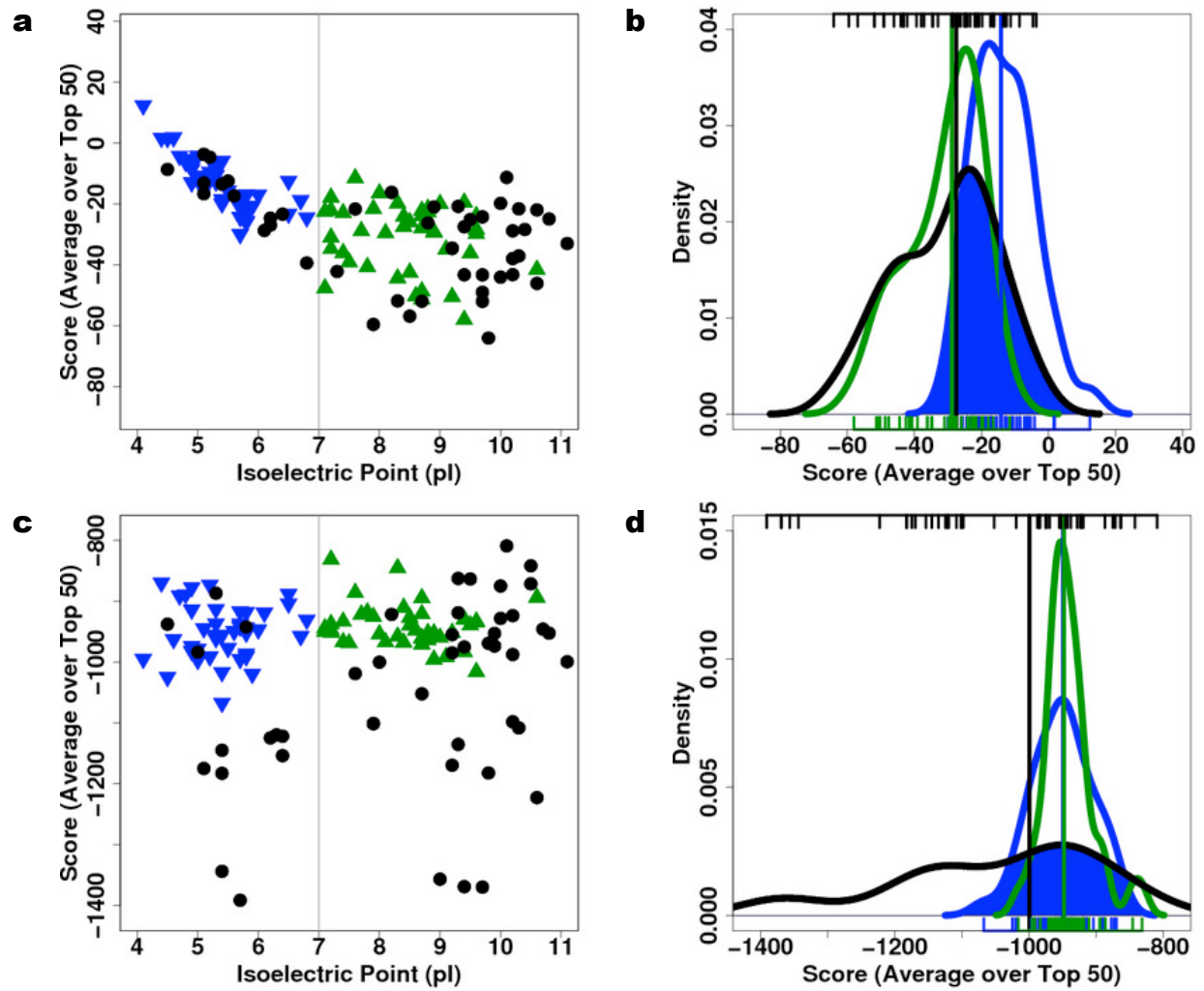

Figure S1

Supplement: Figure S1 — Performance of various scoring functions. The scoring functions are FTdock's Coulomb's law (a,b) and Surface Complementarity Score (c,d). Three decoy sets are used: known DNA-binding proteins (•, black), proteins with pI<7 (▿, blue) and >7 (▵, green). Left panes show how the protein-DNA complexes are scored in relation to the isoelectric point of the protein. Right panes show how the scores of the three decoy sets overlap (blue area) with one another; a perfect scoring function would separate the scores of authentic DNA-binding proteins from those that do not bind DNA. (PDF) [file pone.0032647.s001.pdf]

**a**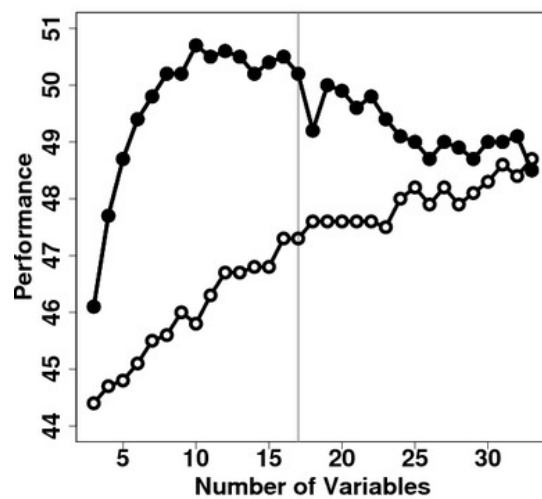**b**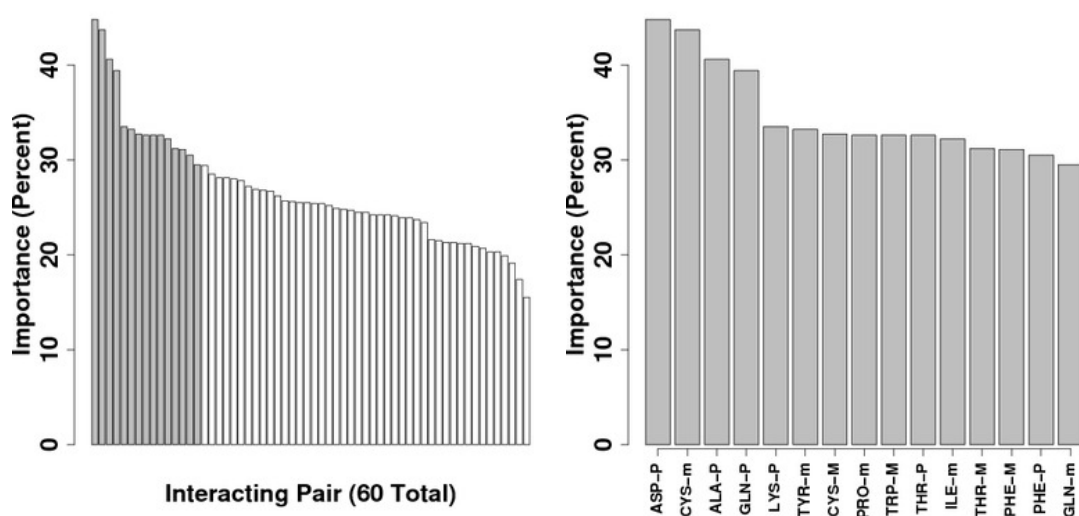

Figure S2

Supplement: Figure S2 — Forward sequential feature selection. The method is applied to find the most important interacting pairs. (a) As the number of variables increases, the performance on the training set (open circles) also increases, but the performance on the test set (closed circles) degrades when too many variables are used; this is the learn-by-heart phenomenon. We thus cap the number of variables to 17 (vertical grey bar); since two of the variables are fixed (Coulomb and |CCP|), 15 will be picked from the 20×3 = 60 interaction matrix. (b) The relative importance of the 60 pairs (left). The 15 most important pairs are highlighted in grey (right). (PDF) [file pone.0032647.s002.pdf]
