# Supplementary material for: On Docking, Scoring and Assessing Protein-DNA Complexes in a Rigid-Body Framework
Source: PLoS One. 2012 Feb 29;7(2):e32647. doi: 10.1371/journal.pone.0032647 (PMC3290582; doi:10.1371/journal.pone.0032647)
Supplement: Table S1 — List of PDB codes that are part of the N = 47 protein-DNA benchmark database. (PDF) [file pone.0032647.s003.pdf]

1a74	1f4k	1pt3	1zme
1azp	1fok	1qne	1zs4
1b3t	1g9z	1qrv	2c5r
1bdt	1h9t	1r4o	2fio
1by4	1hjc	1rpe	2fl3
1cma	1jj4	1rva	2irf
1ddn	1jt0	1tro	2oaa
1dfm	1k79	1vas	3bam
1diz	1kc6	1vrr	3cro
1ea4	1ksy	1w0t	4ktq
1emh	1mnn	1z63	7mht
1eyu	1o3t	1z9c	

Table S1
