# Supplementary material for: On Docking, Scoring and Assessing Protein-DNA Complexes in a Rigid-Body Framework
Source: PLoS One. 2012 Feb 29;7(2):e32647. doi: 10.1371/journal.pone.0032647 (PMC3290582; doi:10.1371/journal.pone.0032647)
Supplement: Table S2 — List of PDB codes that are part of the N = 34 protein-DNA training set database. (PDF) [file pone.0032647.s004.pdf]

1a74	1f4k	1pt3
1azp	1fok	1qne
1b3t	1g9z	1qrv
1bdt	1h9t	1r4o
1by4	1hjc	1rpe
1cma	1jj4	1rva
1ddn	1jt0	1tro
1dfm	1k79	1vas
1diz	1kc6	1vrr
1ea4	1ksy	1w0t
1emh	1mnn	
1eyu	1o3t	

Table S2
