# Supplementary material for: On Docking, Scoring and Assessing Protein-DNA Complexes in a Rigid-Body Framework
Source: PLoS One. 2012 Feb 29;7(2):e32647. doi: 10.1371/journal.pone.0032647 (PMC3290582; doi:10.1371/journal.pone.0032647)
Supplement: Table S3 — List of PDB codes that features N = 40 proteins with isoelectric point lower than 7, and are assumed to not bind DNA. (PDF) [file pone.0032647.s005.pdf]

1ayo-A	1ekj-A	1j77-A	1rhc-A
1d2z-B	1em8-B	1jnd-A	1u8v-A
1dc1-A	1epa-A	1jos-A	1vjz-A
1dek-A	1esw-A	1k4n-A	1xmx-A
1dkq-A	1fp1-D	1kap-P	1ynf-A
1dqp-A	1fye-A	1lc0-A	2c12-A
1dqz-A	1h32-A	1oyg-A	2cdu-A
1e0c-A	1hx6-A	1p1x-A	2dsk-A
1ecs-A	1iap-A	1qop-B	2q4g-W
1ej8-A	1iuq-A	1r0m-A	2yqc-A

Table S3
