# Supplementary material for: On Docking, Scoring and Assessing Protein-DNA Complexes in a Rigid-Body Framework
Source: PLoS One. 2012 Feb 29;7(2):e32647. doi: 10.1371/journal.pone.0032647 (PMC3290582; doi:10.1371/journal.pone.0032647)
Supplement: Table S4 — List of PDB codes that features N = 41 proteins with isoelectric point greater than 7, and are assumed to not bind DNA. (PDF) [file pone.0032647.s006.pdf]

16vp-A	1jy1-A	1qcs-A	2iw1-A
1b7g-O	1jzt-A	1ro2-A	2jlq-A
1btk-A	1l8r-A	1ru4-A	2nt0-A
1d3g-A	1lm8-V	1vky-A	2q62-A
1dow-A	1mnn-A	1wly-A	2qgm-A
1eg3-A	1mww-A	1x9i-A	2zsj-A
1eyb-A	1nep-A	2b3f-A	3bnj-A
1fo8-A	1ofl-A	2b4v-A	3hsi-A
1i4j-A	1oyz-A	2bem-A	
1j1n-A	1p4x-A	2dxu-A	
1jb7-A	1q40-B	2g64-A	

Table S4
