# Supplementary material for: On Docking, Scoring and Assessing Protein-DNA Complexes in a Rigid-Body Framework
Source: PLoS One. 2012 Feb 29;7(2):e32647. doi: 10.1371/journal.pone.0032647 (PMC3290582; doi:10.1371/journal.pone.0032647)
Supplement: Table S5 — Amino acid extent beyond the atom. Values extracted from 201 protein-DNA complexes. The magnitude reported is the average extent plus one standard deviation . The PDB atom types used for the extent measurements are also shown. (PDF) [file pone.0032647.s007.pdf]

AA	Atom	<e> (Å)	AA	Atom	<e> (Å)
ALA	CB	0.00	LEU	CD1	2.53
ARG	NH1	5.28	LYS	NZ	5.07
ASN	OD1	2.42	MET	CE	3.87
ASP	OD1	2.42	PHE	CZ	4.33
CYS	SG	1.84	PRO	CG	1.52
GLN	OE1	3.42	SER	OG	1.48
GLU	OE1	3.37	THR	OG1	1.47
GLY	-	0.00	TRP	CH2	5.41
HIS	CE1	3.65	TYR	OH	5.72
ILE	CD1	2.61	VAL	CG1	1.54

Table S5
