# Supplementary material for: On Docking, Scoring and Assessing Protein-DNA Complexes in a Rigid-Body Framework
Source: PLoS One. 2012 Feb 29;7(2):e32647. doi: 10.1371/journal.pone.0032647 (PMC3290582; doi:10.1371/journal.pone.0032647)
Supplement: Table S6 — Performance of rigid body docking. The performance is measured on a benchmark comprising 47 different complexes. Five docking trials using various combinations of bound and unbound states of the docked molecules: bound/bound (B/B), bound, but with rebuild side chains/bound (S/B), unbound/bound (U/B), bound/unbound (B/U), and unbound/unbound (U/U). A total of 105 docked conformations are generated for each complex and docking trial. For each decoy set, decoys are sorted by RMSD from lowest to greatest, and the RMSD of the 20th best decoy is reported. The RMSD is taken between the DNA positions in the native and the docked complexes, once the proteins have been superimposed. All values reported here are in Angstroms, and are plotted in Figure 1b. The Level column partitions the complexes into the Easy/Intermediate/Difficult notation assigned by van Dijk and Bonvin, which estimates the degree of conformational change upon docking. (PDF) [file pone.0032647.s008.pdf]

PDB	B/B	S/B	U/B	B/U	U/U	Level
1A74	5.67	5.98	4.76	14.37	14.87	Intermediate
1AZP	2.48	2.29	5.41	9.18	7.67	Intermediate
1B3T	3.94	14.08	23.46	22.72	12.43	Difficult
1BDT	3.06	2.97	8.48	9.02	9.86	Difficult
1BY4	3.33	3.28	13.14	6.45	9.14	Easy
1CMA	3.87	7.35	9.20	8.76	6.51	Intermediate
1DDN	4.86	21.27	21.22	19.44	18.70	Intermediate
1DFM	3.35	18.56	24.73	13.68	21.97	Difficult
1DIZ	3.29	3.42	7.93	8.22	7.81	Easy
1EA4	3.20	6.15	7.71	8.68	9.44	Intermediate
1EMH	4.59	4.80	6.05	5.46	6.53	Easy
1EYU	3.29	3.57	9.40	19.32	5.76	Difficult
1F4K	2.79	2.46	13.39	12.88	7.63	Intermediate
1FOK	4.02	4.35	8.62	12.48	12.59	Easy
1G9Z	3.81	7.55	18.04	19.09	10.38	Intermediate
1H9T	4.91	5.01	3.86	14.12	12.52	Easy
1HJC	2.60	3.81	6.64	5.05	6.35	Easy
1JJ4	3.32	7.01	7.79	10.59	10.09	Intermediate
1JT0	6.32	13.35	18.29	17.12	12.81	Intermediate
1K79	2.36	5.15	17.24	17.39	10.26	Intermediate
1KC6	3.16	3.20	11.09	29.99	27.34	Intermediate
1KSY	7.87	5.04	7.01	7.34	4.39	Easy
1MNN	3.64	7.65	10.83	10.49	5.10	Easy
1O3T	6.06	9.74	13.16	14.84	12.42	Difficult
1PT3	2.77	3.89	8.97	9.76	6.52	Easy
1QNE	2.51	2.58	3.04	9.19	10.00	Intermediate
1QRV	2.93	2.92	14.27	10.15	12.86	Difficult
1R4O	2.45	6.05	11.20	11.44	5.89	Intermediate
1RPE	2.42	2.69	4.58	3.98	5.71	Easy
1RVA	3.51	20.55	22.62	21.88	21.22	Difficult
1TRO	3.07	3.16	8.14	9.23	5.99	Easy
1VAS	2.41	3.79	3.64	7.93	7.88	Intermediate
1VRR	3.32	3.31	18.41	17.03	10.17	Intermediate
1W0T	3.17	3.63	10.58	6.10	12.09	Intermediate
1Z63	10.79	10.91	19.91	20.44	10.57	Intermediate
1Z9C	3.32	7.40	4.35	5.07	11.71	Intermediate
1ZME	3.49	3.52	21.78	18.27	14.64	Difficult
1ZS4	3.84	6.04	22.25	21.39	14.59	Intermediate
2C5R	9.70	9.01	11.28	9.76	9.54	Easy
2FIO	11.79	12.13	18.83	19.84	11.64	Intermediate
2FL3	2.97	3.02	14.39	19.68	3.39	Difficult
2IRF	2.37	2.28	11.01	8.64	5.24	Intermediate
2OAA	2.92	3.01	11.79	20.55	5.47	Difficult
3BAM	3.59	3.71	20.58	18.50	15.23	Difficult
3CRO	2.45	2.84	4.25	3.89	3.91	Easy
4KTQ	3.92	8.15	11.89	13.48	10.13	Intermediate
7MHT	3.40	6.53	6.47	12.10	7.42	Difficult
Average	4.02	6.37	11.95	13.08	10.31	

Table S6
