# Supplementary material for: On Docking, Scoring and Assessing Protein-DNA Complexes in a Rigid-Body Framework
Source: PLoS One. 2012 Feb 29;7(2):e32647. doi: 10.1371/journal.pone.0032647 (PMC3290582; doi:10.1371/journal.pone.0032647)
Supplement: Table S7 — Performance of various scoring functions. The performance is tested using the bound states of the protein/DNA molecules. The scoring functions are our work (CCP), contact only statistical potential (C-S) and distance-dependent statistical potential (D-S). The RMSD column is the same as in table S6, B/B case. All values are in Angstroms, and are plotted in Figure 4a. (PDF) [file pone.0032647.s009.pdf]

PDB	RMSD	CCP	D-S	C-S	Level
1A74	5.67	1.43	1.43	22.59	Intermediate
1AZP	2.48	2.38	1.24	18.73	Intermediate
1B3T	3.94	1.43	1.43	1.43	Difficult
1BDT	3.06	1.32	1.32	1.32	Difficult
1BY4	3.33	1.51	1.46	14.66	Easy
1CMA	3.87	26.50	34.31	28.83	Intermediate
1DDN	4.86	1.79	1.68	1.83	Intermediate
1DFM	3.35	3.41	4.24	3.49	Difficult
1DIZ	3.29	28.26	42.01	36.55	Easy
1EA4	3.20	1.24	1.24	1.24	Intermediate
1EMH	4.59	27.47	8.43	28.63	Easy
1EYU	3.29	3.56	1.71	3.39	Difficult
1F4K	2.79	1.55	1.55	31.54	Intermediate
1FOK	4.02	43.12	1.79	5.89	Easy
1G9Z	3.81	1.19	1.19	4.50	Intermediate
1H9T	4.91	1.66	1.66	1.76	Easy
1HJC	2.60	8.02	8.02	2.39	Easy
1JJ4	3.32	1.27	1.27	25.10	Intermediate
1JT0	6.32	1.32	1.32	6.24	Intermediate
1K79	2.36	1.74	2.27	2.76	Intermediate
1KC6	3.16	3.80	3.71	3.80	Intermediate
1KSY	7.87	1.52	15.21	45.66	Easy
1MNN	3.64	1.70	1.70	1.70	Easy
1O3T	6.06	5.84	1.42	5.93	Difficult
1PT3	2.77	3.10	23.87	26.38	Easy
1QNE	2.51	1.59	1.59	33.18	Intermediate
1QRV	2.93	33.39	1.67	27.74	Difficult
1R4O	2.45	1.82	1.94	14.76	Intermediate
1RPE	2.42	1.80	1.53	24.59	Easy
1RVA	3.51	1.96	3.49	5.45	Difficult
1TRO	3.07	1.70	1.70	38.38	Easy
1VAS	2.41	1.28	1.28	26.25	Intermediate
1VRR	3.32	3.49	6.07	8.28	Intermediate
1W0T	3.17	1.79	1.85	2.91	Intermediate
1Z63	10.79	47.91	43.26	39.02	Intermediate
1Z9C	3.32	1.72	1.72	12.70	Intermediate
1ZME	3.49	1.40	1.40	47.00	Difficult
1ZS4	3.84	1.47	1.47	29.79	Intermediate
2C5R	9.70	14.35	24.82	13.97	Easy
2FIO	11.79	1.46	32.92	14.37	Intermediate
2FL3	2.97	1.59	2.95	22.53	Difficult
2IRF	2.37	2.74	1.63	8.63	Intermediate
2OAA	2.92	3.01	1.45	31.68	Difficult
3BAM	3.59	8.15	1.70	1.57	Difficult
3CRO	2.45	1.28	1.28	1.33	Easy
4KTQ	3.92	1.71	4.86	3.87	Intermediate
7MHT	3.40	1.63	1.63	40.94	Difficult
Average	4.02	6.67	6.55	16.50	

Table S7
