# Supplementary material for: On Docking, Scoring and Assessing Protein-DNA Complexes in a Rigid-Body Framework
Source: PLoS One. 2012 Feb 29;7(2):e32647. doi: 10.1371/journal.pone.0032647 (PMC3290582; doi:10.1371/journal.pone.0032647)
Supplement: Table S8 — Summary of Tables S6 and S7, grouped by van Dijk and Bonvin's annotations. A checkmark is put whenever the DNA RMSD is lower than 10 Angstroms. The FFT column relates to the ability of the FFT-based docking method to provide for good decoys, while the last three columns to the ability of the various scoring functions to identify them. Rows marked with an asterix have been left out while training, and as such constitute the test set. (PDF) [file pone.0032647.s010.pdf]

PDB	FFT	CCP	D-S	C-S
Easy				
*2C5R	✓			
1PT3	✓	✓		
1MNN	✓	✓	✓	✓
1FOK	✓		✓	✓
1KSY	✓	✓		
*3CRO	✓	✓	✓	✓
1EMH	✓		✓	
1H9T	✓	✓	✓	✓
1TRO	✓	✓	✓	
1BY4	✓	✓	✓	
1HJC	✓	✓	✓	✓
1DIZ	✓			
1RPE	✓	✓	✓	
Intermediate				
1VRR	✓	✓	✓	✓
1F4K	✓	✓	✓	
1K79	✓	✓	✓	✓
1KC6	✓	✓	✓	✓
1EA4	✓	✓	✓	✓
*1Z63				
1R4O	✓	✓	✓	
1AZP	✓	✓	✓	
1W0T	✓	✓	✓	✓
1CMA	✓			
1JJ4	✓	✓	✓	
1VAS	✓	✓	✓	
*4KTQ	✓	✓	✓	✓
*1Z9C	✓	✓	✓	
1DDN	✓	✓	✓	✓
*2IRF	✓	✓	✓	✓
1JT0	✓	✓	✓	✓
1G9Z	✓	✓	✓	✓
1A74	✓	✓	✓	
*2FIO		✓		
1QNE	✓	✓	✓	
*1ZS4	✓	✓	✓	
Difficult				
1QRV	✓		✓	
1O3T	✓	✓	✓	✓
1B3T	✓	✓	✓	✓
*3BAM	✓	✓	✓	✓
1RVA	✓	✓	✓	✓
*1ZME	✓	✓	✓	
1DFM	✓	✓	✓	✓
1BDT	✓	✓	✓	✓
*7MHT	✓	✓	✓	
*2FL3	✓	✓	✓	
1EYU	✓	✓	✓	✓
*2OAA	✓	✓		

Table S8
